# The key players of inflammasomes and pyroptosis in sepsis-induced pathogenesis and organ dysfunction

**DOI:** 10.3389/fphar.2025.1586364

**Published:** 2025-05-19

**Authors:** Lujian Zhu, Minli Hu, Haoming Xu, Hanglu Xu, Binbin Ren, Ruiyan Xu, Maodong Guo, Haijun Chen, Dehe Zhang, Hao Fang

**Affiliations:** ^1^ Department of Infectious Disease, Affiliated Jinhua Hospital, Zhejiang University School of Medicine, Jinhua, China; ^2^ Department of Gastroenterology, Affiliated Jinhua Hospital, Zhejiang University School of Medicine, Jinhua, China; ^3^ Department of Geriatric Respiratory, HeBei Provincial General Hospital, Shijiazhuang, China; ^4^ Department of Clinical Skills Center, Affiliated Jinhua Hospital, Zhejiang University School of Medicine, Jinhua, China; ^5^ Department of Trauma Surgery, Affiliated Jinhua Hospital, Zhejiang University School of Medicine, Jinhua, China

**Keywords:** pyroptosis, NLRP3 inflammasome, caspase-1, sepsis, organ dysfunction

## Abstract

Pyroptosis is an inflammatory form of cell death involving caspase-1 or caspase-4/5/11, initiated by inflammasomes or cytoplasmic endotoxins as part of the immune defense. It is specifically characterized by Gasdermin-mediated pore formation leading to cell lysis, pyroptosis also entails the release of pro-inflammatory cytokines. As a natural mechanism of the immune system, it activates in response to harmful stimuli to eliminate threats and facilitate tissue repair. However, excessive pyroptosis can lead to detrimental outcomes, such as infectious shock, multiple organ dysfunction syndrome (MODS), and increased susceptibility to secondary infections. Sepsis, an unchecked immune response to infection, remains a leading cause of MODS and death among critically ill patients. The pathogenesis of sepsis is complex and multifaceted, involving innate inflammation that kills infected cells and releases pro-inflammatory cytokines. Recent research has increasingly explored the link between pyroptosis and sepsis, focusing on its mechanisms, roles, and potential therapeutic targets. There has been significant advancement in understanding pyroptosis, highlighting its vital role in the development of sepsis. This review delves into the molecular and pathophysiological roles of inflammasomes and pyroptosis in sepsis, with a particular emphasis on the impact on specific organs such as the heart, lungs, liver, kidney and brain, aiming to identify new diagnostic markers and therapeutic targets for sepsis management.

## Introduction

Sepsis is a systemic inflammatory response triggered by an infection that leads to a dysregulated immune response. This common issue can stem from various severe injuries, burns, infections, extensive surgeries, compromised immune systems, advanced cancers, etc (Fernanda et al., 2021; Xue et al., 2022). Sepsis can lead to MODS or even death, with mortality rates increasing as more organs are affected (Lichao et al., 2009).

From data published in 2020, there were 48.9 million cases and 11 million sepsis-related deaths worldwide, representing 20% of all global deaths (Kristina et al., 2024). Sepsis, along with its related organ failure remains a leading cause of death in intensive care units globally, posing a significant threat to life and health (Tatiana et al., 2022). In the field of critical illness, sepsis and multiorgan injury from infections present substantial challenges. Current treatments primarily focus on symptomatic relief and combating infections. However, understanding the molecular mechanisms of sepsis is crucial for developing new therapeutic targets. The complexity of sepsis revolves around the regulation of inflammatory and immune responses (Jie et al., 2021). Recent studies suggest a pivotal role for pyroptosis in these processes, particularly during sepsis. In the early stages of sepsis, the body activates its immune defense and triggers programmed cell death to combat the infection (Swathy et al., 2023). Pyroptosis, a specific type of programmed cell death, is integral to the innate immune response. It prevents the replication of intracellular pathogens and stimulates immune cells to engulf and eliminate pathogens, thus protecting the host from bacterial and microbial infections (Larissa and Dario, 2013; Megan et al., 2021; Pian et al., 2021). However, excessive pyroptosis can amplify the inflammatory response in surrounding cells and tissues, exacerbating inflammation and potentially leading to organ failure or septic shock (Yuan-Yuan et al., 2018).

Pyroptosis is an inflammatory form of cell death characterized by pore formation in cell membranes, leading to cell swelling, rupture, and the release of large amounts of inflammatory mediators such as IL-1β and IL-18 (Charles et al., 2021; Yi et al., 2021). Central to this process is the inflammasome, which plays a pivotal role in mediating interactions between the immune system and cellular components (Esteban et al., 2023). As a key element of the innate immune system, the inflammasome is vital for defending the body against pathogens and external stressors by activating caspase-1 and stimulating the release of inflammatory cytokines like IL-1β and IL-18 (Yi et al., 2021). The inflammasome also plays a crucial role in the body’s response to pathogenic threats. Its activation is essential for the innate immune response upon pathogen infection (Nijin et al., 2023). Caspase-1, a critical component of the inflammasome, serves a protective function against various pathogenic invasions (Hila et al., 2020). Additionally, recent studies have highlighted the role of Caspase-11 in the noncanonical inflammasome pathway, which is involved in pyroptosis and the release of specific inflammatory molecules (Kevin et al., 2020; Xu et al., 2023). This underscores that the inflammasome is integral not only to intracellular signaling and regulation but also to the cellular response to external stimuli. This review explores the involvement of the inflammasome and pyroptosis in sepsis and its associated organ dysfunction, with a particular emphasis on the impact on specific organs such as the heart, lungs, liver, kidney and brain, trying to provide new perspectives on potential treatments for sepsis and related organ complications in a clinical context.

## Inflammasome and pyroptosis

Pyroptosis is an innate immune mechanism in animals that influences homeostasis and aging, characterized by an inflammatory form of programmed cell death. This process is initiated by inflammatory caspases to guard against external pathogens (Xiang et al., 2022). To understand pyroptosis, it's essential to recognize a critical component: the “inflammasome.” Tschopp and his team (Fabio et al., 2002) first coined the term “inflammasome” to describe a complex assembly of protein structures that detect internal threats using NOD-like receptors (NLRs). NLRs are part of the pattern recognition receptor family and feature a C-terminal leucine repeat sequence for ligand recognition, a central NACHT domain responsible for oligomerization and dNTPase activation, and an N-terminal domain (Shraddha and Thirumala-Devi, 2020). Based on the NACHT structural domains, researchers have classified the protein complex into three main subgroups: 1) NOD (including NOD1-5, CIITA); 2) NLRP or NALP (comprising NLRP/NALP1-14); and 3) IPAF (consisting of IPAF, NAIP) (Reza et al., 2014). Additionally, there are various classifications based on N-terminal effector domains, and not all NLRs contribute to the formation of inflammasomes. With the exception of AIM2, the inflammasome is named based on NLRs (Tomasz et al., 2016).

To date, researchers have identified four main types of inflammasomes: NLRP1 (NALP1), NLRP3 (NALP3), NLRC4 (IPAF), and AIM2. Each type has unique ligand recognition sites and utilizes different adapter molecules, but all are capable of activating caspase-1, which initiates an inflammatory response (Jianing and Hao, 2023; V et al., 2013; Wen-Juan et al., 2021). Among these, the NLRP3 inflammasome is particularly significant in the human immune system, primarily regulating both inflammation and pyroptosis (Jing et al., 2022). It consists of three components: the NLRP3 sensing protein, the ASC protein with a CARD domain, and a precursor of caspase-1 (Congjian et al., 2020; Xiao-Feng et al., 2018), which come together to form a complex that was initially identified and named by Hoffman and colleagues (Davide et al., 2024).

The activation of the NLRP3 inflammasome occurs through one of three main pathways: potassium ion efflux, the production of reactive oxygen species (ROS), or lysosomal damage that leads to rupture (Cai-Song et al., 2023). In the first pathway, specific stimuli such as bacterial toxins or ATP prompt the P2X7 purinergic receptor to detect these external signals, which in turn activates a potassium channel on the cell membrane. This leads to the efflux of potassium ions from the cell and the buildup of ubiquitylated connexins, ultimately triggering the activation of NLRP3. NLRP3 then forms a pore in the membrane, allowing external pathogen-associated molecular patterns (PAMPs) and damage-associated molecular patterns (DAMPs) to enter the cytosol (Feng et al., 2021; Yanzhao et al., 2022). A decrease in potassium ion levels serves as a key stimulus for activating the NLRP3 inflammasome, although the detailed mechanism behind this activation remains somewhat elusive. In the second pathway, an important role is played by lysosomes, which are intracellular digestive organs that contain a variety of enzymes used to break down substances from both internal and external cellular sources (Eleazar et al., 2020; Zhang R. et al., 2022). The ingestion of substantial amounts or sizes of particulate matter, such as uric acid crystals, cholesterol crystals, or asbestos fibers, can result in lysosomal damage and rupture (Seungwha et al., 2021). When lysosomes rupture, they release enzymes and other materials that can either directly or indirectly trigger the activation of the NLRP3 inflammasome (Yan et al., 2021). In the third pathway, ROS has been found to initiate NLRP3 activation. ROS, which include peroxides and free radicals, are produced during normal cellular metabolism and play a role in cellular signaling at low levels but can cause cellular damage when elevated (Min et al., 2022; Shasha et al., 2020). A number of stimuli, such as infection or cellular stress, can lead to excessive production of ROS (David et al., 2022). The NLRP3 inflammasome is particularly sensitive to increases in ROS, which are believed to be a significant trigger for its activation (Junyuan et al., 2023). Approaches to inhibit NLRP3 activation include reducing ROS production or using NADPH oxidase inhibitors or scavengers that eliminate ROS (Hai-Yang et al., 2023). Although ROS are produced by various cellular stresses and can be induced by large particulate matter and ATP, their presence alone does not necessarily result in NLRP3 activation (Ilandarage Menu Neelaka et al., 2019).

NLRP3 inflammasome activation is a complex process influenced by various pathways, notably potassium ion efflux, ROS production, lysosomal damage, etc. Potassium efflux is recognized as a critical trigger for NLRP3 inflammasome activation, typically occurring in response to various stimuli, including ATP and pore-forming toxins, leading to a decrease in intracellular potassium levels. The reduction in potassium concentration is necessary for the assembly of the NLRP3 inflammasome and subsequent activation of caspase-1. ROS is also a key link involved in the activation of the NLRP3 inflammasome. Mitochondrial dysfunction often leads to increased ROS levels, which can facilitate NLRP3 assembly by enhancing the interaction between NLRP3 and its activators. Additionally, it is reported that ROS production can be influenced by potassium efflux, indicating an interconnected relationship between these two pathways. Lysosomal integrity is crucial for preventing inappropriate NLRP3 activation, lysosomal damage contributes to NLRP3 activation by releasing cathepsins and other DAMPs into the cytosol. Hence, the above pathways play distinct yet interrelated roles in the activation of the NLRP3 inflammasome, understanding these interactions is essential for developing targeted therapies aimed at modulating inflammasome activity in various inflammatory diseases.

Recent studies have shown that males and females exhibit different patterns of inflammasome activation, which may affect their susceptibility to sepsis and the severity of organ dysfunction. It has been identified that estrogen can ameliorate some diseases such as sepsis, Parkinson’s disease, inflammatory bowel disease, spinal cord injury, multiple sclerosis, myocardial ischemia/reperfusion injury, and renal fibrosis, by inhibiting the NLRP3 inflammasome. Conversely, estrogen can also promote the development of diseases including ovarian endometriosis, dry eye disease, and systemic lupus erythematosus by upregulating the NLRP3 inflammasome (Wanglin et al., 2023). However, the mechanism of these effects is not summarized. Adu-Amankwaah et al. illuminated that sepsis-induced cardiac dysfunction and mortality are more pronounced in males than females, they revealed that estradiol, acting via the G protein-coupled estrogen receptor 1 (GPER-1), enhances cardiac function and metabolism while reducing oxidative stress and apoptosis in females during sepsis. Additionally, GPER-1 activation in males mirrors these benefits, improving cardiac function and survival rates, suggesting GPER-1 as a potential therapeutic target for sepsis treatment. Incorporating insights from other recent studies about sex discrepancy will provide a more comprehensive understanding of how sex differences impact pyroptosis in sepsis (Joseph et al., 2024; Kristy et al., 2024; Martin et al., 2013; Vera et al., 2023).

### The two main molecular mechanisms of pyroptosis

Pyroptosis operates through two primary molecular pathways: the classical and non-classical (Zhan-Fei, 2023). Both pathways culminate in the release of the pro-inflammatory cytokines IL-1β and IL-18, which further enhance both local and systemic inflammatory responses (Yao W. et al., 2023) (Table 1).

**TABLE 1:** Summary of major inflammasomes and functions.

Inflammasome type	Pathway activation	Caspase activation	Cytokine production	Associated diseases
NLRP1	Activated by various PAMPs and DAMPs	Caspase-1	IL-1β, IL-18	Autoimmune diseases, infections (Adam and Kerrie, 2020)
NLRP3	Potassium ion efflux, ROS production, lysosomal damage	Caspase-1	IL-1β, IL-18	Sepsis, metabolic disorders (Agudelo-Ochoa et al., 2020)
NLRC4	Recognizes bacterial flagellin	Caspase-1	IL-1β	Bacterial infections (Akiko et al., 2022)
AIM2	Detects cytosolic DNA from pathogens	Caspase-1	IL-1β	Viral infections, autoimmune diseases (Austin and Mariola, 2021)

### The classical pathway of pyroptosis

The classical pyroptosis pathway, a tightly regulated mode of cell death, that focuses on the critical role of inflammatory vesicles. In this process, the cleavage of Gasdermin D (GSDMD) and the subsequent release of IL-1β and IL-18 are key events (Xia et al., 2019).

**FIGURE 1:**
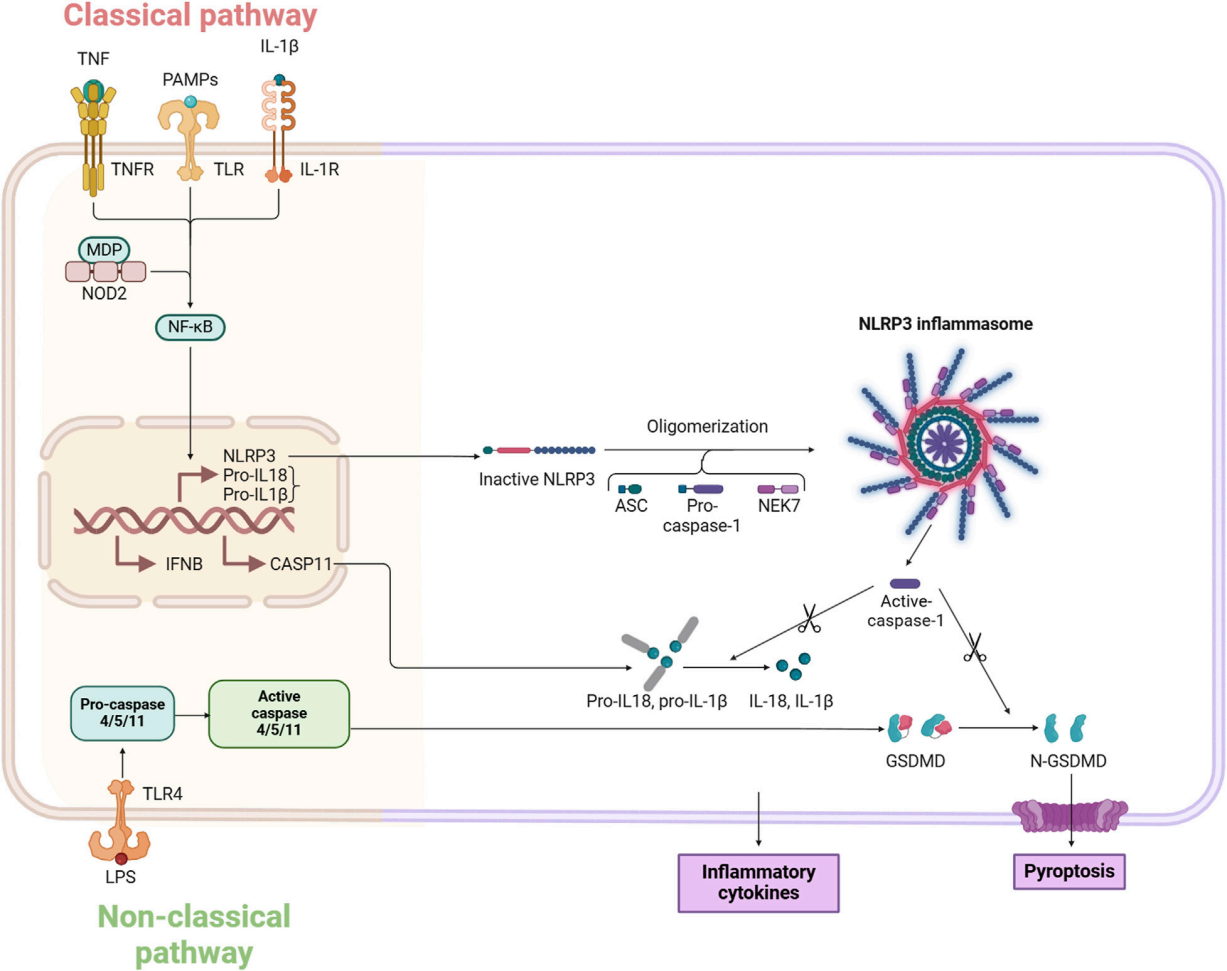
Molecular mechanisms of pyroptosis.

In the classical pyroptosis pathway, the NLRP3 inflammasome plays a central role, activated by the detection of various PAMPs and DAMPs (Yan et al., 2022; Yeyu et al., 2022). NLRP3 is a well-studied inflammasome that can be triggered by a range of stimuli including bacteria, viruses, moisture, and pore-forming toxins (Marta et al., 2022). Upon stimulation by these pyroptosis-inducing agents, inflammasomes quickly assemble and activate caspase-1 (Xinzhang et al., 2022). The activation of caspase-1 leads to the cleavage of GSDMD, producing an active N-terminal GSDMD fragment (Yuanyuan et al., 2021). This fragment can insert into the cell membrane to form a pore, resulting in the leakage of cellular contents and cell swelling (Ning et al., 2022). Additionally, the activation of caspase-1 results in the processing of IL-1β and IL-18 precursors, leading to the production of mature IL-1β and IL-18. These mature cytokines are then expelled from the cell, amplifying the inflammatory response. This pathway is thus also referred to as the caspase-1-dependent pyroptosis pathway (Lizda et al., 2023) (Figure 1).

### Non-classical pyroptosis pathways

The non-classical pyroptosis pathway is predominantly triggered by caspase 4/5/11, rather than caspase-1 (Shi-Wei et al., 2023). In humans, caspase-4 and caspase-5 are primarily involved, while caspase-11 is found in mice (Chin King et al., 2021). In this non-canonical pathway, the caspase recruitment domain (CARD) of caspase-4/5/11 binds to lipopolysaccharide (LPS), promoting its own clustering and activation, which leads to enzyme activity (Yan-Yang et al., 2019). Once activated, caspase-4/5/11 cleaves GSDMD into its active form, GSDMD-NT, on the cell membrane, forming pores approximately 10–15 nm in size. GSDMD also stimulates the activation of the caspase-1-dependent NLRP3 inflammasome, contributing to the release of IL-1β and IL-18 and indirectly enhancing the canonical pyroptosis pathway (Jingjing et al., 2022; Liyan et al., 2023; Xiaohua et al., 2023). Pyroptosis is crucial in producing IL-1β and IL-18, leading to both acute and chronic inflammation, and it also acts as an endogenous pyrogen, playing a role in fever development.

### The role of pyroptosis in sepsis and its associated organ dysfunction

Pyroptosis plays a pivotal role in the progression of sepsis, particularly in the transition from localized infection to systemic inflammation and multiorgan dysfunction. Initially, upon infection, the immune system activates pyroptosis as a defense mechanism to eliminate intracellular pathogens. This process is characterized by the formation of pores in infected cells through GSDMD cleavage, leading to cell lysis and the release of pro-inflammatory cytokines such as IL-1β and IL-18. These cytokines are crucial for recruiting immune cells to the site of infection, thereby enhancing local inflammation and promoting pathogen clearance. However, if the infection is not adequately controlled, the excessive activation of pyroptosis can lead to a dysregulated inflammatory response (Bhargavi and Christian, 2019). The release of large amounts of inflammatory mediators into circulation can result in systemic inflammation, characterized by widespread activation of immune cells and increased vascular permeability. This systemic response can overwhelm the body’s regulatory mechanisms, leading to MODS. As sepsis progresses, the continuous cycle of pyroptosis and inflammation contributes to tissue damage across various organs, including the lungs, kidneys, and heart. The severity of organ dysfunction correlates with the degree of pyroptosis activation, highlighting its role as both a protective mechanism and a contributor to detrimental outcomes in sepsis (Ruifei et al., 2022; Ruoyu et al., 2024). During the initial phases of sepsis, while it may damage tissues, controlled pyroptosis serves as a protective mechanism that helps eradicate pathogens and prevent the spread of infections (Zhi-Ying et al., 2022). Upon infection, the host triggers the activation of immune cells like macrophages to engage in the innate immune response (Xi et al., 2020). Macrophages are key in these responses, primarily through the release of IL-1β and IL-18 (Tongchao et al., 2019). However, as sepsis progresses, excessive pyroptosis can lead to an uncontrolled inflammatory response, significantly worsening the course and outcome of sepsis, and contributing to a grim prognosis (Hao et al., 2022).

**FIGURE 2:**
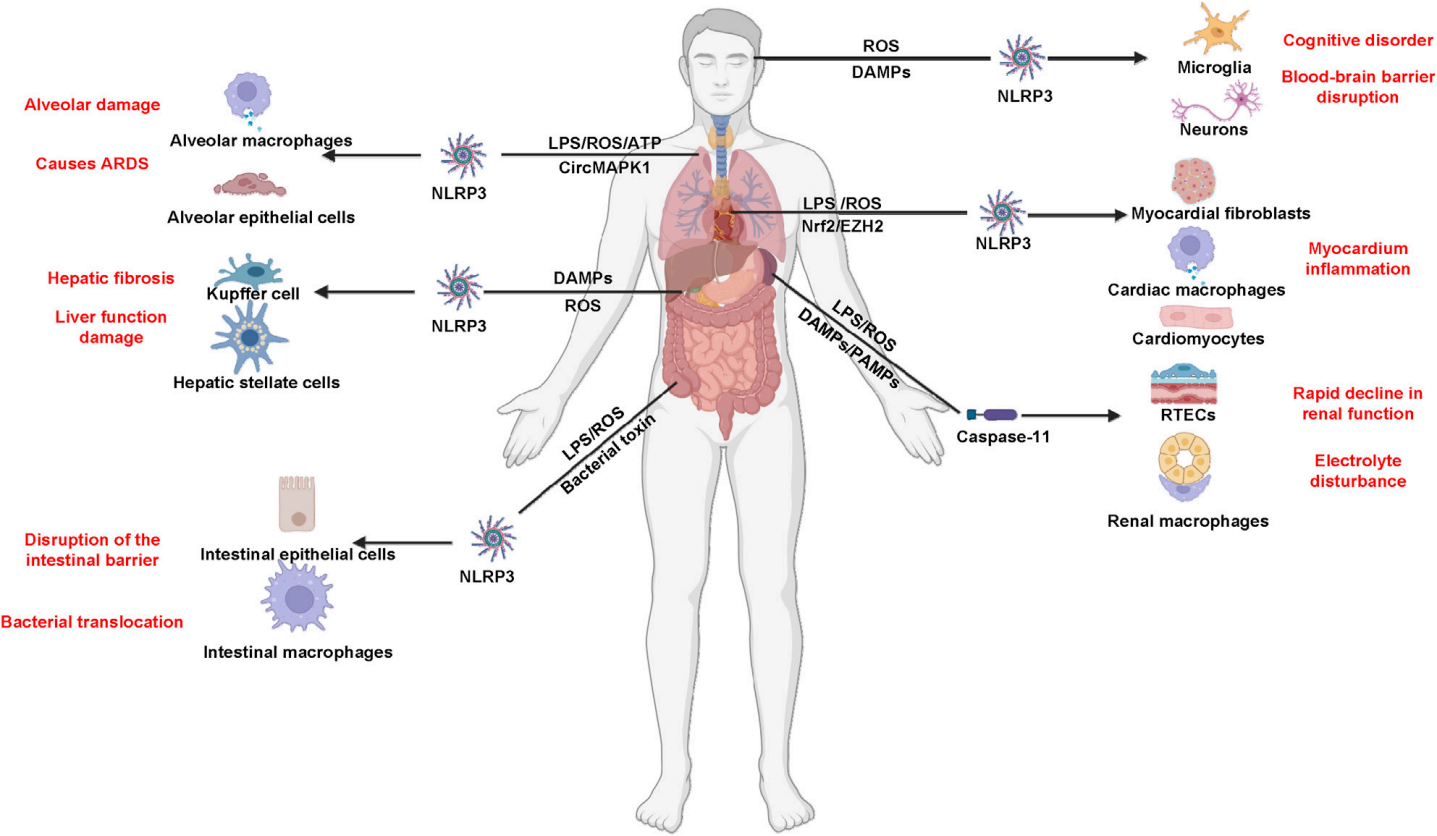
Inflammasome and pyroptosis in sepsis and sepsis-associated MODS.

The gastrointestinal tract plays a critical role in the pathophysiology of sepsis, serving both as a target and a source of inflammation. Disruption of the gut barrier can lead to increased intestinal permeability, allowing for bacterial translocation into the bloodstream. This process not only exacerbates systemic inflammation but also contributes significantly to the development of MODS. In a healthy state, the gut barrier functions to prevent the passage of pathogens and toxins into systemic circulation. However, during sepsis, factors such as ischemia, hypoxia, and inflammatory mediators can compromise this barrier. The resulting increase in intestinal permeability facilitates the translocation of bacteria and their products, such as LPS, into the bloodstream. This bacterial translocation triggers an overwhelming immune response, characterized by the release of pro-inflammatory cytokines that can lead to widespread inflammation and tissue damage across various organs. The inflammatory response initiated by gut-derived bacteria can activate pathways such as pyroptosis, further amplifying systemic inflammation. Pyroptosis in intestinal epithelial cells contributes to local tissue damage and enhances the inflammatory milieu, which can spill over into systemic circulation. This vicious cycle not only worsens sepsis but also heightens the risk of developing MODS. Strategies aimed at preserving gut barrier function, such as probiotics or targeted anti-inflammatory therapies, could mitigate bacterial translocation and its sequelae. Furthermore, identifying biomarkers related to gut integrity and inflammation may aid in early diagnosis and intervention. Above all, the comprehensive understanding these mechanisms is essential for developing targeted therapies that can modulate pyroptosis and mitigate its harmful effects while preserving its protective functions (Figure 2).

### Pyroptosis-mediated organ dysfunction associated with sepsis

Recent studies have highlighted the crucial role of pyroptosis in the onset of multiple organ dysfunctions linked to sepsis (Ri et al., 2022). The severity of post-sepsis tissue damage and multiple organ dysfunction syndromes is proportional to the severity of pyroptosis. Interestingly, inhibitors to curtail pyroptosis can reduce the inflammatory response, thereby attenuating the damage of sepsis-induced multiple organ dysfunction (Yi-Hao et al., 2020). The intracellular inflammatory responses and pyroptosis, which vary across different organs, have been extensively investigated for their underlying mechanisms in recent years. However, the effectiveness of pyroptosis inhibitors has primarily been tested using non-human experimental models, indicating a need for further clinical validation (Table 2).

**TABLE 2:** Organ-specific effects of pyroptosis in sepsi**s**.

Organ	Effect of pyroptosis	Experimental model	Mechanism	Key findings
Lungs	Increased inflammation leading to acute respiratory distress syndrome (ARDS)	LPS-induced ALI model in mice; LPS/ATP- stimulated macrophage model	Pyroptosis in alveolar macrophages releases IL-1β and IL-18, exacerbating lung inflammation	NLRP3 inhibition reduces IL-18 and lung inflammation (Bhargavi and Christian, 2019)
Heart	Sepsis-induced myocardial injury (SIMI) characterized by impaired ventricular function	CLP mouse model; LPS-stimulated cardiomyocyte/macrophage models	Pyroptosis contributes to cardiomyocyte inflammation and damage, leading to cardiac dysfunction	GSDMD and NLRP3 mediate myocardial damage (Agudelo-Ochoa et al., 2020; Bhat et al., 2024)
Liver	Hepatic dysfunction and increased susceptibility to liver injury	*E. coli* bloodstream infection model in mice; NLRP3 and GSDMD knockout mice	Pyroptosis in liver macrophages leads to excessive cytokine release, promoting liver inflammation	GSDMD knockout mice show improved survival and less liver damage (Agudelo-Ochoa et al., 2020; Bhat et al., 2024)
Kidneys	Acute kidney injury (AKI) due to inflammatory cell infiltration	LPS-induced sepsis model in mice; zebrafish model using lethal LPS dose	Pyroptosis in renal tubular cells enhances inflammatory responses, leading to tubular damage	Caspase-11 and GSDME mediate tubular injury (Blutt et al., 2023; Brown et al., 2013)
Intestines	Disruption of gut barrier function and increased gut permeability	CLP-induced intestinal injury in mice	Pyroptosis affects intestinal epithelial cells, facilitating translocation of bacteria and toxins	Inflammasome activation leads to bacterial leakage and MODS (Cai-Song et al., 2023; Carissa and Edward, 2022)
Brain	Neuroinflammation and potential development of septic encephalopathy	CLP mouse model for SAE; *in vitro* brain tissue/mitochondrial oxidative stress assays	Pyroptosis in microglia leads to the release of pro-inflammatory cytokines affecting neuronal function	NLRP3 activation worsens ROS and BBB disruption (Carolin et al., 2015; Charles et al., 2021)

### Pyroptosis and sepsis-induced myocardial injury (SIMI)

SIMI, which stems from cardiovascular complications associated with sepsis, is characterized by temporary enlargement of the left ventricle and impaired ventricular function during systole and/or diastole (Song et al., 2023). Inflammation affects cardiomyocytes, leading to infiltration, edema, and mitochondrial damage, although necrosis of cardiomyocytes does not occur, providing a potential for mitigating myocardial damage during sepsis (Jianling et al., 2019). Patients with sepsis who also develop SIMI face a worse prognosis, with significantly increased morbidity and mortality rates. Echocardiography and biomarkers are increasingly utilized for clinical detection of SIMI (Yatong et al., 2022). While specific diagnostic criteria for SIMI are lacking, research has connected various cellular processes like apoptosis, autophagy, pyroptosis, and necrosis to the condition (Yanjing et al., 2022), yet the precise mechanisms remain unknown. The initiation of the inflammatory response plays a vital role in SIMI (Lin et al., 2022), triggering the release of numerous pro-inflammatory factors such as TNF-α, IL-6, and IL-1β during sepsis. Angiotensin II among other factors stimulate macrophage activity (Fuyun et al., 2019), with IL-1β playing a central role in the inflammatory process in sepsis-induced cardiac issues (Ryo et al., 2020).

Recently, increasing evidence has established a connection between inflammation, pyroptosis, and the progression of SIMI. The development of sepsis is influenced by oxidative stress, apoptosis, and inflammation, which are regulated under specific conditions and affect the progression of scorosis (Lingling et al., 2020). The molecular mechanisms of pyroptosis include components such as inflammatory vesicles, members of the caspase family, interleukins, and GSDMD (Xiao-Qiong et al., 2022). Targeting these molecules can help reduce the severity of heart disease. Studies on the pathophysiology of pyroptosis in sepsis have mainly focused on the GSDMD and NLRP3-mediated signaling pathways (Yongsheng et al., 2023). The ER/SIRT1/NLRP3/GSDMD signaling pathway, which is the most comprehensive pathway currently identified, also influences the mechanism of pyroptosis in SIMI. GSDMD plays a crucial role in programmed cell death, with specific caspases such as caspase-4, 5, and caspase-11 activating it to trigger pyroptosis (Xiang-Hou et al., 2023). This mechanism is similarly observed in sepsis. Additionally, pyroptosis, a form of cell death involving an inflammatory response mediated by GSDMD, highlights the importance of NLRP3 inflammatory vesicles as critical signaling molecules in pyroptosis within SIMI. Recent research has shown that rhodopsin offers a protective effect on the heart, confirming that the activation of NLRP3 inflammatory vesicles contributes to cardiomyocyte death during sepsis (Zhange et al., 2020). Zhang et al. demonstrates that sepsis-induced activation of the NLRP3 inflammasome/caspase-1/IL-1β pathway in cardiac fibroblasts contributes to myocardial dysfunction, and that inhibition of this pathway improves cardiac function and survival in septic mice (Zhang et al., 2014). The regulation of this process by NLRP3 is influenced by caspase-1, ROS, and NF-κB signaling pathways (Yanyan et al., 2022). CTRP1 serves as a promoter that prevents cellular proximity, and its function is activated by interacting with increased levels of Nrf2 (Sumei et al., 2023), a critical regulator of cellular defense mechanisms against stress. This interaction protects cells from damage by controlling the activation of various protective genes (Chun-Yan et al., 2020). In sepsis, diminished Nrf2 levels lead to the inactivation of the CTRP1 binding site, causing cardiomyocyte pyroptosis (Yi et al., 2020). Additionally, studies have shown that LPS activation elevates NLRP3 levels through the interaction of the stimulator of interferon genes (STING) with type I interferon regulatory factor 3 (IRF3), leading to IRF3 phosphorylation (Weizhe et al., 2020). Deleting STING reduces LPS-induced SIMI in mice (Lingling et al., 2023).

Bai et al. (Xue et al., 2024) reported the potential interaction of LncRNA and pyroptosis, they found that LncRNA SOX2OT mitigates sepsis-induced myocardial injury by suppressing pyroptosis through the EZH2/Nrf-2/NLRP3 signaling pathway, conversely, SOX2OT knockdown exacerbated LPS-induced levels of inflammatory factors and procalcitonin, and increased the expression of pyroptosis-related proteins and lactate dehydrogenase. These findings delve into the genetic mechanisms of septic shock and could pave the way for new approaches to prevent and treat systemic inflammatory response syndrome.

### Pyroptosis and sepsis-associated lung injury

Acute lung injury (ALI) is a common complication of sepsis, characterized by damage to alveolar epithelial and endothelial cells, infiltration of inflammatory cells into the lungs, and symptoms of congestion and swelling (Nan et al., 2020). The development of infectious ALI is associated with inflammation, oxidative stress, and regulated cell death mechanisms. Increasing evidence suggests a link between pyroptosis and sepsis-induced ALI (Yu-Chang et al., 2019). The NLRP3/Caspase-1/GSDMD pathway, a key inflammatory pathway, plays a critical role in the pathogenesis of ALI, with the resulting alveolar macrophage pyroptosis being a primary contributor to lung damage and pulmonary (Huayu et al., 2022). Recent studies have shown that inhibiting NLRP3 activation in macrophages can reduce the release of IL-1β and IL-18, curb inflammation, and alleviate tissue damage in septic mice (Yi-Fu et al., 2023). Macrophage pyroptosis plays a crucial role as an inflammatory mechanism in sepsis-induced lung injury, Li et al. (Min et al., 2024) identified that CircMAPK1 was elevated in patients with septic lung injury, and knockdown of circMAPK1 protected against LPS/ATP-impaired cell viability and macrophage pyroptosis via WNK1/NLRP3 axis. They confirmed that CircMAPK1 exacerbates sepsis-induced lung injury by destabilizing KDM2B mRNA to suppress WNK1 expression, thus facilitating NLRP3-driven macrophage pyroptosis. Moreover, blocking Caspase-1-mediated pyroptosis could markedly diminish the organism’s excessive inflammatory response, thereby improving ALI and reducing the LPS-induced inflammatory response in the pulmonary vascular endothelial cells and alveolar macrophages of septic mice (Jie Y. et al., 2022). In contrast, ARDS represents the most severe and frequent form of lung injury related to sepsis and is a major contributor to the prevalence and mortality of sepsis, with reported rates ranging from 30% to 45% among hospitalized patients (Carolin et al., 2015). Current Western medical treatments primarily focus on addressing the underlying disease and providing respiratory support, yet there remains a notable lack of effective drugs and methods to achieve optimal therapeutic outcomes. The use of antibiotics and glucocorticoids in the clinical management of sepsis is often associated with significant side effects and the risk of drug dependency. Therefore, the development of drugs with therapeutic potential for the treatment of ALI is of great significance.

### Pyroptosis and sepsis-associated liver injury

The liver, the largest glandular organ in the body, is vital for maintaining metabolic and immune equilibrium due to its extensive blood supply, and it is frequently compromised during sepsis. The precise mechanisms behind acute liver damage in sepsis are not fully understood, though many studies suggest links to oxidative stress and excessive inflammatory responses (Bingtao et al., 2022). Liver injury often occurs in the early stages of sepsis and significantly influences the body’s ability to clear bacteria or LPS, serving as a critical indicator of sepsis outcomes (Yao et al., 2022). The liver plays a crucial role in protecting against pathogens entering the bloodstream. In addition, prior Studies have shown that hepatocytes, Kupffer cells, and hepatic stellate cells (HSC) engage in a pathway sensitive to pyroptosis, which can trigger cell death through the detection of PAMPs or DAMPs or lead to liver damage through intercellular connections (Jérémie et al., 2020).

The significance of inflammatory vesicle activation and pyroptosis in hepatocytes contributing to liver damage should not be overlooked. A recent study revealed that proteins linked to the pyroptosis pathway were markedly elevated in patients with acute liver injury during sepsis, while mice with reduced expression of GSDMD exhibited less liver damage and significantly improved survival (Jie W. et al., 2022). This animal infection model lays the groundwork for exploring the impact of hepatocyte pyroptosis on liver injury in sepsis. In experiments involving *E. coli* bloodstream infections, mice deficient in NLRP3 demonstrated higher survival rates and less inflammatory cell infiltration in tissues compared to their wild-type counterparts. Moreover, NLRP3 was shown to increase the severity and mortality rates of widespread bacterial infections, independently of IL-1β and IL-18 (Yanwen et al., 2020). The authors suggested that excessive activation of NLRP3 may lead to the release of endogranulinic acid (Eicosanoid) through the pyroptosis pathway, which could intensify sepsis (Austin and Mariola, 2021). Additionally, Kupffer cells, which are specialized macrophages in the liver capable of self-renewal, play an essential role in preserving immune balance by absorbing endotoxins and releasing inflammatory molecules. These cells are the primary immune constituents in the liver’s microenvironment and are a significant source of IL-1β, IL-18, and NLRs (Valentina et al., 2019). Experiments conducted both in a laboratory setting and in living organisms demonstrated a notable increase in the levels of NLRP3 and IL-1β expression in Kupffer cells in a sepsis model induced by LPS, highlighting their strong response to the pyroptosis pathway (Sergei et al., 2023). In contrast, HSCs are endothelial pericytes located in the perisinusoidal space of the liver, which transform into myofibroblasts and produce extracellular matrix during liver injury, playing a role in post-injury repair. The activation of HSC is also to the onset and development of hepatic fibrosis (Shuang et al., 2023; Suwei et al., 2021). Studies have found that in a mouse model of sepsis, the activation of inflammatory vesicles in HSC leads to increased levels of connective tissue growth factor (CTGF) and tissue inhibitor of matrix metalloproteinase 1 (TIMP1), contributing to increased collagen deposition and hepatic fibrosis in liver tissues (Akiko et al., 2022).

### Pyroptosis and sepsis-associated acute kidney injury (SA-AKI)

SA-AKI typically manifests early in the course of sepsis, marked by a rapid and sustained decrease in kidney function over a short period, often escalating to acute kidney failure or even death. The kidneys are frequently affected by sepsis, with the incidence of SA-AKI increasing annually (Yoon et al., 2024). Many patients with infectious shock experience acute renal failure early on. During systemic infections, LPS from Gram-negative bacteria plays a critical role in activating cellular pyroptosis, making it a significant factor in inducing septic shock (Carissa and Edward, 2022). In the context of sepsis, both DAMPs and PAMPs enter the bloodstream. The glomerulus can filter out small, low molecular weight DAMPs and PAMPs. However, proximal tubular epithelial cells that come into contact with DAMPs and PAMPs undergo oxidative stress, leading to the production of ROS and mitochondrial damage. Infiltration of inflammatory cells induces cellular autophagy and mitochondrial dysfunction in renal tubular epithelial cells. This condition leads these cells to lose their cell polarity and undergo apoptosis and necrosis, ultimately reducing the glomerular filtration rate and causing renal injury (Pierre-Olivier et al., 2021). Renal tubular epithelial cells are the primary contributors to kidney injury, with inflammation playing a key role in the progression of SA-AKI. Research has shown that inhibiting caspase-11 reduces pyroptosis-related protein levels in renal tubular epithelial cells (RTECs), suggesting that blocking caspase-11 could help prevent RTECs from undergoing pyroptosis (Kai et al., 2023; Zhiming et al., 2019). Furthermore, a study employing zebrafish as a model organism used a bacterial mutant library and high-throughput screening technology to activate a fish cell death model and identify critical inflammatory caspases that regulate cell death in fish. Building on this research, another study developed a reverse genetic screening approach for experimental fish using CRISPR/Cas9 technology. This rapid screening method discovered that the expression of caspase-11 in RTECs could be inhibited. It also found that deleting the Caspy2 and GSDMEb genes could mitigate septic shock in zebrafish exposed to a lethal dose of LPS. Structural characterization, subcellular localization, and the role of GSDME proteins in the innate immune signaling pathway demonstrated that activated Caspy2 can cleave GSDME proteins. This cleavage leads to the multimerization of the GSDMEb N-terminal fragment and perforation of the cell membrane, causing pyroptosis in fish cells (Zhuang et al., 2020). These studies provide potential therapeutic targets and strategies for managing and treating SA-AKI.

### Pyroptosis and sepsis-associated encephalopathy (SAE)

SAE, a common neurodegenerative condition in sepsis, affects approximately 70% of septic patients. Its pathogenesis includes neuroinflammation, changes in neuronal function and signaling, cerebral hyper-perfusion resulting from microcirculatory issues, oxidative stress, and disruptions in the blood-brain barrier (Ha-Yeun et al., 2023; Teneille and G Bryan, 2012). This condition is typically a systemic response to infection, rather than a direct CNS infection. Clinically, it manifests in a spectrum from mild malaise and attention deficits to severe coma, and is associated with high morbidity and mortality, leading to a grim prognosis. Studies indicate that cellular pyroptosis intersects with various pathological processes that contribute to SAE, playing a significant role in brain damage. This includes compromising the blood-brain barrier, inducing oxidative stress, damaging mitochondria, and disrupting brain metabolism (Milan et al., 2023). Cellular pyroptosis is a key factor in the progression of SAE, underscoring the importance of preventing neuronal pyroptosis to enhance treatment outcomes and prognosis in SAE. Recent studies have shown that in a mouse model of sepsis induced by cecal ligation and puncture (CLP), caspase-1 inhibitors effectively reduced cell death, decreased the release of inflammatory molecules, and preserved the brain’s ultrastructure. These actions collectively improved SAE conditions and protected cognitive functions in mice (Xi et al., 2019). In the initial stages of sepsis, the brain often undergoes alterations such as oxidative stress, reduced energy production, and impaired cerebral metabolism. Prior research has demonstrated that the ratio of superoxide dismutase to peroxidase activity is significantly elevated in the brains of rats with sepsis (Tatiana et al., 2006). Furthermore, animal studies have verified that oxidative stress leads to mitochondrial damage and that mitigating oxidative stress can decrease brain damage in mice with sepsis (Hui-Ru et al., 2023).

Cellular physiological functions are significantly influenced by ROS originating from mitochondria. It was discovered that an excessive increase in ROS production initiates the formation of the NLRP3 inflammasome and enhances GSDMD cleavage, leading to the initiation of pyroptosis (Fenghong et al., 2022). Concurrently, it was observed that the NLRP3 inflammasome may amplify the release of mitochondrial ROS, and inhibiting NLRP3 can reduce the mitochondrial oxidative stress response (ShiChun et al., 2020), suggesting that there may be a mutual regulatory effect between NLRP3 inflammasome and mitochondrial ROS. Wang et al. (Miao et al., 2024) found that YL-109, a novel compound, could mitigate sepsis-associated multi-organ injury by inhibiting the ERK/AP-1 axis and pyroptosis, and upregulating CHIP expression, demonstrating that YL-109 protects against LPS-induced high mortality, cardiac dysfunction, and pulmonary and intestinal injuries in mice, offering insights into its protective mechanisms against organ damage. In summary, oxidative stress can trigger pyroptosis, and managing the oxidative stress pathway could suppress pyroptosis, potentially serving as a critical target for SAE treatment (Table 3).

**TABLE 3:** Inhibitors targeting pyroptosis in sepsis and MOD**S**.

Inhibitor	Target	Mechanism	Model	Administration method	Observed effects
Z-VAD-FMK	Pan-caspases	Broad-spectrum caspase inhibitor blocking IL-1β/IL-18 releases	CLP in rats/mice	Intraperitoneal injection	Reduces IL-1β/IL-18 releases; improves survival (Chen et al., 2023; Chin King et al., 2021)
VX-765	Caspase-1	Selective inhibition of caspase-1 activity	LPS-induced sepsis model	Oral gavage	Attenuates inflammation and organ injury (Chopyk and Grakoui, 2020)
MCC950	NLRP3	Prevents NLRP3 inflammasome assembly via ATPase inhibition	CLP model in mice	Intraperitoneal injection	Reduces inflammasome activation; mitigates tissue damage (Chen et al., 2023; Chin King et al., 2021)
Necrosulfonamide	GSDMD	Binds GSDMD Cys191, blocks membrane pore formation	LPS-stimulated macrophages	*In vitro*	Prevents pyroptosis in macrophages (Chopyk and Grakoui, 2020)
YL-109	CHIP/ERK/AP-1 axis	Reduces pyroptosis by modulating ERK signaling	LPS-induced multiple organ injury in mice	Intraperitoneal injection	Protects against cardiac, pulmonary, and intestinal damage (Charles et al., 2021)
PEITC	GSDMD	Direct inhibition of GSDMD to reduce hepatocyte pyroptosis	Acute liver injury model in mice	Intraperitoneal injection	Alleviates liver damage; improves survival (Bingtao et al., 2022)

## Implications of targeted pyroptosis in the treatment of sepsis and its associated organ dysfunction

While advancements in medical technology have reduced the morbidity and mortality associated with sepsis, it remains a significant cause of death due to infectious shock and sepsis. Sepsis is predominantly triggered by infections that cause prolonged excessive inflammation followed by immunosuppression. Identifying more effective therapeutic targets is essential. Pyroptosis, which is involved in both inflammation and immune modulation, presents new possibilities for sepsis management. Initial studies by Braun et al. (1999), Hotchkiss et al. (1999) found that the broad-spectrum caspase inhibitor Z-VAD-FMK provided protection against pneumococcal meningitis in a New Zealand rat model and a mouse model of sepsis. Subsequent research showed that in septic patients suffering from endotoxemia and *Staphylococcus aureus* infections, Z-VAD-FMK significantly reduced IL-1β release by blocking caspase activity. Moreover, the comprehensive caspase inhibitor VX-166 demonstrated potent anti-pyroptotic and anti-inflammatory effects, significantly improving therapeutic outcomes in sepsis by reducing the release of IL-1β and IL-18 in rats using a CLP model (Lijun et al., 2023). In addition to broad-spectrum inhibitors, more selective inhibitors targeting pyroptosis effectors have shown promise. For instance, VX-765, a selective caspase-1 inhibitor, has been demonstrated to attenuate inflammation and organ injury in an LPS-induced sepsis model through oral gavage administration (Wen et al., 2022). Similarly, MCC950, a specific inhibitor of the NLRP3 inflammasome, effectively prevents inflammasome assembly by inhibiting its ATPase activity, and has been shown to reduce inflammasome activation and tissue damage in a CLP-induced sepsis mouse model via intraperitoneal injection (Tapia-Abellan et al., 2019). While these agents show promise in preclinical models for reducing excessive inflammation and tissue damage, their broad-spectrum inhibition raises concerns about potential immunosuppressive effects that could compromise the body’s ability to combat infections. Therefore, research should focus on developing more selective inhibitors that can modulate pyroptosis without broadly suppressing immune responses, involving targeting specific components of the inflammasome or downstream signaling pathways. Additionally, inhibiting GSDMD may be beneficial for treating sepsis in clinical environments. Necrosulfonamide, a GSDMD inhibitor, has been shown to prevent NLRP3-induced pyroptosis in mouse macrophages by attaching to Cys191 on GSDMD in a sepsis model, which resulted in improved survival rates in murine sepsis (Jacob et al., 2020). Of note, balancing the reduction of pyroptosis-related damage with the preservation of vital immune functions is essential for optimizing patient outcomes.

At present, several clinical trials are performed to evaluate the safety and efficacy of the target genes. For instance, it is reported that the NLRP3 inhibitors and caspase inhibitors may play a protective role in organ function via regulating inflammatory markers in septic patients (Adam and Kerrie, 2020; Ildiko et al., 2022). These trials aim to provide insights into optimal dosing regimens and identify patient populations that may benefit most from pyroptosis-targeting therapies. While targeting pyroptosis presents a promising strategy for mitigating organ dysfunction in sepsis, challenges remain in clinical translation. Current therapies, such as NLRP3 inflammasome inhibitors and caspase-1 blockers, may partly suppress necessary immune responses, leading to increased susceptibility to infections and impaired tissue repair mechanisms. Additionally, the variability in pyroptosis effects across different organs complicates the development of universally effective treatments. The risk of immunosuppression and the potential for exacerbating organ dysfunction highlight the need for careful patient selection and monitoring in clinical settings. In summary, molecules involved in sepsis-related pyroptosis could serve as potential diagnostic and prognostic markers, and importantly, as therapeutic targets. Although these inhibitors have not yet been implemented in clinical practice, further research is essential to better understand pyroptosis mechanisms and to develop effective treatments for sepsis in clinical settings.

### The role of the intestinal microbiota in organ dysfunction mediated by inflammasome activation and pyroptosis during sepsis

Recent studies have highlighted the critical role of the intestinal microbiota in modulating immune responses during sepsis, particularly through its influence on inflammasome activation and pyroptosis (Haak and Wiersinga, 2017; Man, 2018). The gut microbiota not only shapes host immunity under homeostatic conditions but also plays a pivotal role in the pathogenesis of MODS during systemic inflammatory responses (Schuijt et al., 2013). Destruction of intestinal microbiome predisposes to sepsis, and has a negative impact on the results of sepsis (Agudelo-Ochoa et al., 2020; Fay et al., 2017; Shreiner et al., 2015). The gut microbiota influences sepsis-induced MODS primarily through intestinal barrier disruption, microbial metabolite signaling, and perturbation of gastrointestinal immune homeostasis (Brown et al., 2013; Chopyk and Grakoui, 2020; Song et al., 2020; Zhao et al., 2023). Chen et al. identified a pregnancy-associated reduction of the gut bacterium *Parabacteroides merdae*, which leads to decreased formononetin levels and enhanced NLRP3 inflammasome-mediated pyroptosis, thereby increasing susceptibility to sepsis (Chen et al., 2023). Another study demonstrated that sepsis-induced gut microbiota dysbiosis contributes to susceptibility to SAE, and that the microbiota-derived metabolite indole-3-propionic acid (IPA) alleviates neuroinflammation by inhibiting NLRP3 inflammasome activation in microglia (Fang et al., 2022). Tang et al. reported that exercise-induced gut microbiota remodeling, particularly enrichment of *Ligilactobacillus* and its metabolite betulinic acid, protects against sepsis-induced acute liver injury by suppressing NLRP3 inflammasome activation via the hnRNPA2B1 pathway (Tang et al., 2025). Gut microbiota dysbiosis promotes age-related atrial fibrillation via LPS- and glucose-induced NLRP3 inflammasome activation, suggesting a potential role of the gut microbiota in SIMI (Zhang Y. et al., 2022). Elucidating the molecular mechanisms by which the gut microbiota regulates inflammasome activity is a key focus of future research and may play a vital role in developing therapeutic strategies for sepsis-induced MODS. For example, one study demonstrated that gut microbiota-derived metabolites such as taurine, histamine, and spermine regulate intestinal homeostasis by modulating NLRP6 inflammasome activity and antimicrobial peptide expression, and disruption of this axis leads to dysbiosis and colitis (Levy et al., 2015).

### Systematic multiomics to explore the role of inflammasome and pyroptosis in sepsis mediated MODS

Multi-omics approaches—including genomics, epigenomics, transcriptomics, proteomics, metabolomics, and immunomics—offer comprehensive insights into the mechanisms underlying sepsis-induced MODS (Blutt et al., 2023; Levy et al., 2015; Qiao and Cui, 2022). Using multi-omics approaches, including transcriptomics, metabolomics, and microbiome analysis, researchers reveal that GSDMD plays a protective role in ConA-induced autoimmune hepatitis. The absence of GSDMD led to worsened liver injury, increased inflammation, gut barrier disruption, and gut microbiota dysbiosis, suggesting that pyroptosis contributes to maintaining immune and gut-liver homeostasis during inflammation (Wang et al., 2022). Recent multi-omics studies have provided valuable insights into the immune-metabolic dysregulation associated with sepsis-induced MODS, though direct evidence linking inflammasome activation and pyroptosis remains limited. Li et al. integrated proteomic, transcriptomic, and metabolomic profiling of serum exosomes from septic patients, revealing that exosomes regulate IL-1β, IL-6, and other pro-inflammatory cytokines—key downstream effectors of inflammasome activation. These vesicles were shown to exacerbate cytokine storms and organ injuries such as acute kidney injury via mechanisms involving proteasome-mediated protein degradation and vitamin metabolism, implicating a potential role of inflammasome-related signaling in systemic metabolic disturbances (Li et al., 2022). Complementarily, Tong et al. applied Mendelian randomization to combined eQTL and pQTL datasets, identifying inflammation-related genes such as IL18R1 and C5 as genetic regulators of sepsis susceptibility and 28-day mortality. Given the known associations of these genes with inflammasome pathways, these findings suggest possible links between genetic regulation of pyroptosis-related processes and MODS pathogenesis (Tong et al., 2025). The researchers extensively discussed the application and significance of transcriptomics, miRNomics, and circRNA-omics in elucidating the mechanisms of pyroptosis and sepsis-induced organ dysfunction. They highlighted that various non-coding RNAs (ncRNAs) regulate the activation of inflammasomes (e.g., NLRP3), caspases, and Gasdermin D-mediated pore formation, thereby promoting the release of pro-inflammatory cytokines such as IL-1β and IL-18. These regulatory pathways play critical roles in the pathogenesis of sepsis-induced organ injuries, including AKI, myocardial injury, and ALI (Bhat et al., 2024). In the future, the application of multi-omics techniques will serve as a powerful approach to uncover the key regulatory mechanisms of inflammasome activation and pyroptosis, offering critical insights into the pathogenesis of sepsis-induced MODS.

## Summary and prospects

This review highlights the latest discoveries regarding the development of pyroptosis, its key regulatory mechanisms, and its role in causing organ damage during sepsis. Researchers increasingly recognize sepsis as a complex condition involving various protective and harmful pathways, rather than just an uncontrolled inflammatory response. Pyroptosis plays a vital role in the immune response, significantly impacting the progression and severity of sepsis. Initially, pyroptosis helps prevent the proliferation of pathogens within cells and facilitates their rapid elimination. However, its excessive activation can lead to severe inflammation and organ dysfunction. Although updates to sepsis management guidelines are improving treatment approaches, outcomes remain suboptimal. Therefore, a focused effort on elucidating the mechanisms of sepsis and pyroptosis is essential for developing targeted therapies,the ability to effectively control pyroptosis in sepsis has profound clinical implications, potentially reducing mortality rates and preventing the progression to MODS, which are critical outcomes in the management of sepsis patients. Future research should prioritize identifying specific therapeutic targets within the pyroptotic pathway to mitigate sepsis-induced organ damage and improve treatment efficacy.
